# Applications of Nanoparticles in the Diagnosis and Treatment of Ovarian Cancer

**DOI:** 10.3390/nano15151200

**Published:** 2025-08-06

**Authors:** Ahmed El-Mallul, Ryszard Tomasiuk, Tadeusz Pieńkowski, Małgorzata Kowalska, Dilawar Hasan, Marcin Kostrzewa, Dominik Czerwonka, Aleksandra Sado, Wiktoria Rogowska, Igor Z. Zubrzycki, Magdalena Wiacek

**Affiliations:** 1 Faculty of Medical Sciences and Health Sciences, Radom University, Chrobrego 27, 26-600 Radom, Poland; a.el-mallul@uthrad.pl (A.E.-M.); r.tomasiuk@uthrad.pl (R.T.); aleksandra.sado.1999@gmail.com (A.S.); wiktoriaa.rogowska@gmail.com (W.R.); i.zubrzycki@urad.edu.pl (I.Z.Z.); 2 Libyan Academy for Postgraduate Studies, Sidi Abdoljalil Road 3, Janzur 40225, Libya; 3 The Centre of Postgraduate Medical Education, ul. Marymoncka 99/103, 01-813 Warszawa, Poland; tadeusz.pienkowski@cmkp.edu.pl; 4 Radom Oncology Center, ul. Uniwersytecka 6A, 26-600 Radom, Poland; 5 Faculty of Applied Chemistry, Radom University, Chrobrego 27, 26-600 Radom, Poland; m.kowalska@uthrad.pl (M.K.); m.kostrzewa@urad.edu.pl (M.K.); d.czerwonka@uthrad.pl (D.C.); 6 School of Engineering and Sciences, Technologico de Monterrey, Atizapan de Zaragoza 52926, Estado de Mexico, Mexico; dilawar.hassan1992@gmail.com

**Keywords:** nanoparticles, ovarian cancer, diagnosis, treatment

## Abstract

Nanotechnology offers innovative methodologies for enhancing the diagnosis and treatment of ovarian cancer by utilizing specialized nanoparticles. The utilization of nanoparticles offers distinct advantages, specifically that these entities enhance the bioavailability of therapeutic agents and facilitate the targeted delivery of pharmacological agents to neoplastic cells. A diverse array of nanoparticles, including but not limited to liposomes, dendrimers, and gold nanoparticles, function as proficient carriers for drug delivery. Nevertheless, notwithstanding the auspicious potential of these applications, challenges pertaining to toxicity, biocompatibility, and the necessity for comprehensive clinical evaluations pose considerable barriers to the widespread implementation of these technologies. The incorporation of nanotechnology into clinical practice holds the promise of significantly transforming the management of ovarian cancer, offering novel diagnostic tools and therapeutic strategies that enhance patient outcomes and prognoses. In summary, the deployment of nanotechnology in the context of ovarian cancer epitomizes a revolutionary paradigm in medical science, amalgamating sophisticated materials and methodologies to enhance both diagnostic and therapeutic outcomes. Continued research and development endeavors are essential to fully realize the extensive potential of these innovative solutions and address the existing challenges associated with their application in clinical settings.

## 1. Introduction

This review aims to assess current advancements in nanoparticle-based technologies for the diagnosis and treatment of ovarian cancer, highlighting their clinical potential and existing limitations.

Cancer represents one of the foremost causes of mortality on a global scale [1]. The delayed identification of malignant lesions, coupled with the limited efficacy of conventional therapeutic modalities that exhibit low specificity and considerable toxicity, adversely affects the prognostic outcomes for affected individuals [2,3]. Among various neoplasms, ovarian cancer ranks as one of the principal causes of mortality in the female population [4], and it uniquely represents the sole malignancy of the female reproductive system that may manifest during childhood [4]. The incidence of fatalities attributable to ovarian cancer is primarily a consequence of the nonspecific clinical manifestations that arise during the initial stages of the illness, thereby complicating accurate diagnostic efforts in the later phases of the disease when the chances of patient recovery are exceedingly low. Consequently, the majority of ovarian cancer cases are diagnosed at advanced stages (stage III or IV), at which point the malignancy has typically metastasized to adjacent tissues, thereby diminishing the efficacy of treatment interventions [5,6]. Contemporary diagnostic approaches for ovarian cancer, such as the assessment of tumor markers, including CA-125, as well as imaging modalities like ultrasound, computed tomography (CT), and magnetic resonance imaging (MRI), when employed during the early stages of the disease, lack sufficient sensitivity to enable unequivocal diagnosis [7,8]. Moreover, delays in the diagnosis of ovarian cancer frequently stem from nonspecific detection criteria and the prohibitive costs associated with the employed methodologies [9,10]. Within this context, nanotechnology addresses the need for precise diagnostic capabilities and economically viable therapeutic strategies, offering innovative solutions specifically adapted to confront the challenges prevalent in the battle against ovarian cancer [11,12].

Nanoparticles, characterized as “zero-dimensional” entities, represent materials with dimensions ranging from 1 to 100 nanometers, exhibiting distinctive physical, chemical, and biological characteristics that are markedly different from those of their larger analogs [13]. These nanoparticles operate by selectively interacting with biomarkers found in oncological tissues, such as those present in ovarian cancer cells [14,15]. The interaction of a nanoparticle with a cancer cell can be ascertained through various diagnostic methodologies [12,16] facilitating the early identification of ovarian neoplasms—a pivotal factor in the timely commencement of therapeutic interventions at the initial stages of the disease [16,17].

Furthermore, nanoparticle-mediated drug delivery systems epitomize a pioneering approach for the targeted administration of therapeutic agents to designated anatomical regions within the organism. Their diminutive dimensions facilitate the seamless transgression of biological impediments, including cellular membranes and the blood–brain barrier [18]. Consequently, the application of nanoparticles as drug delivery vehicles enhances the bioavailability of pharmacologically active compounds while mitigating the adverse effects associated with treatment. Moreover, nanoparticles possess the capability to be synthesized in response to specific environmental cues, such as pH variations or thermal changes, thereby facilitating the regulated release of therapeutic agents. Additionally, these particles can undergo modifications with diverse ligands, thereby enabling the targeted delivery to specific cellular phenotypes, including malignant cells [19].

Furthermore, nanoparticles can enhance the bioavailability and localization of biologically active molecules within and around neoplastic tissues, thereby ensuring the delivery of therapeutics at efficacious concentrations [20,21]. The functionalization of nanoparticles with specialized ligands or antibodies facilitates targeted therapeutic interventions, potentially augmenting the efficacy of treatment regimens [22,23,24]. In summary, conventional oncological interventions, such as surgical resection, radiotherapy, and chemotherapy, are associated with considerable adverse effects [25,26], which nanoparticle technology has the potential to mitigate.

Continued advancements in nanotechnology may lead to significant improvements in the management of ovarian cancer, particularly through enhanced drug delivery systems and targeted therapies that minimize side effects [27,28,29,30].

**Figure 1:**
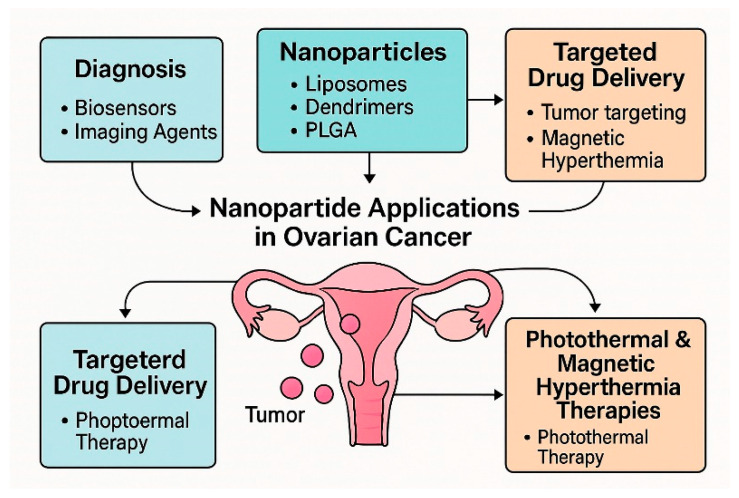
Applications of Nanoparticles in the Diagnosis and Treatment of Ovarian Cancer.

In recent years, there has been a significant increase in scientific research that confirms the effectiveness of nanoparticle applications in the treatment and diagnosis of cancer [31]. Since nanoparticles have numerous applications in medicine, we will limit our review to the literature specifically related to the applications of nanotechnology in the diagnosis and treatment of ovarian cancer. Moreover, we are paying special attention to the potential applications of various nanomaterials and their role in the diagnosis and treatment of ovarian cancer (Figure 1).

In summary, nanoparticles hold great promise for enhancing both the specificity and efficacy of therapeutic interventions in ovarian cancer, ultimately aiming to improve patient survival rates and quality of life [32]. Advancements in nanoparticle technology are expected to continue driving innovations in targeted drug delivery, particularly in the context of ovarian cancer treatment [33]. By enhancing the precision of therapeutic interventions, these technologies may significantly improve patient outcomes and reduce treatment-related side effects. The integration of nanotechnology into ovarian cancer management could lead to breakthroughs in early detection and personalized treatment strategies, ultimately enhancing survival rates and patient quality of life.

## 2. Types of Nanoparticles

**Figure 2:**
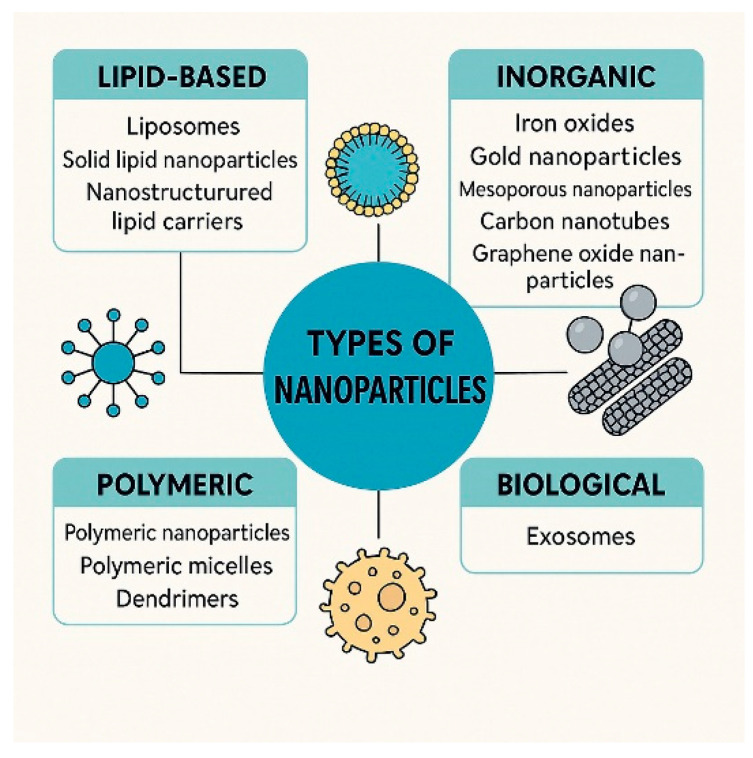
Classification and Key Properties of Nanoparticles Relevant to Ovarian Cancer Applications.

Among different types of nanoparticles, one may distinguish lipid-based, inorganic, polymeric, and biological nanoparticles. Lipid-based nanoparticles comprise liposomes, solid lipid nanoparticles, and nanostructured lipid carriers. Inorganic nanoparticles include iron oxides, gold nanoparticles, mesoporous nanoparticles, carbon nanotubes, and graphene oxide nanoparticles. Within polymeric, it is possible to distinguish polymeric nanoparticles, polymeric micelles, and dendrimers. And within biological nanoparticles, exosomes (Figure 2).

Nanoparticles are defined as particles exhibiting dimensions from 1 to 100 nanometers, characterized by extensive surface areas that enhance their chemical reactivity and specific physical attributes due to the quantum and surface phenomena that are significant at nanoscale [34]. Furthermore, the considerable surface area facilitates effective environmental interactions, which is crucial for the adsorption of various chemicals, biomolecules, or other nanoparticles [18]. Nonetheless, it is essential to note that surface characteristics are not the sole determinant of nanoparticle properties; the geometry of the nanoparticles is equally significant [19]. Nanoparticles can assume diverse morphologies, including spherical or rod-like shapes, aggregate into more intricate structures, and interact with other molecular entities. These distinctive physicochemical characteristics enhance the precision, accuracy, efficiency, and safety associated with drug delivery mechanisms [35]. Among the various classifications of nanoparticles, one can identify iron oxide nanoparticles, metallic gold nanoparticles, silver nanoparticles, semiconductor nanoparticles, organic nanoparticles, carbon-based nanoparticles, silica nanoparticles, and fullerenes.

Metal oxide nanoparticles, such as iron oxide (Fe_3_O_4_), are employed in magnetic resonance imaging to help visualize ovarian tumors [36]. They may also act as carriers for drug delivery to tumor cells, which improves treatment effectiveness [35,37,38]. Gold and silver nanoparticles serve a dual purpose as diagnostic tools and drug delivery systems in the treatment of ovarian cancer. Additionally, gold nanoparticles are utilized in various biomedical imaging methods, including computed tomography (CT) and magnetic resonance imaging (MRI), while also functioning as drug transporters [39,40]. In contrast, silver nanoparticles are recognized for their antibacterial effects and can be applied in cancer treatment [41].

Quantum dots, which are semiconductor nanoparticles, emit bright visible or near-infrared light when subjected to UV light or laser exposure, making them valuable for medical diagnostics, such as imaging cancer cells [41]. Organic nanoparticles, including liposomes and polymeric nanoparticles, can as carriers for chemotherapy drugs and may also function as gene blockers or immunosuppressive agents [42,43,44]. Carbon nanoparticles, particularly carbon nanotubes, possess distinctive electrical and mechanical properties, enabling their use as drug carriers [42,43] and as biosensors to track the progress of therapy [44,45]. Other types of nanoparticles, such as silicas, are utilized in the biomedical field, serving as materials for diagnostic imaging or as coatings that regulate drug release [46]. Fullerenes are being explored as potential therapeutic agents for ovarian cancer and are currently under clinical evaluation [47,48].

In recent years, biogenic nanocarriers have captivated the interest of the scientific community. One of the foremost benefits of using biogenic nanocarriers is their inherent biocompatibility, which minimizes adverse effects when administered to patients. Studies have shown that biogenic nanoparticles, such as those derived from natural sources like plant extracts, can enhance drug efficacy while reducing toxicity [49,50]. These nanoparticles can be functionalized with specific ligands that target ovarian cancer cells, enabling the selective delivery of chemotherapeutic agents directly to the tumor site. Such a targeting capability is critical in ovarian cancer, where minimizing side effects is crucial due to the complex nature of the disease and the treatment regimen involved [51].

A critical component in the development of biogenic nanocarriers is the use of targeting moieties. Recent advancements have demonstrated the effectiveness of using peptides, such as follicle-stimulating hormone (FSH) peptide, as targeting ligands [51,52,53]. FSH receptors are overexpressed in certain ovarian tumors, and utilizing FSH peptide-conjugated nanoparticles has been shown to facilitate enhanced drug delivery specifically to ovarian carcinoma, improving treatment outcomes [52]. In animal models, FSH-conjugated nanoparticles have demonstrated effective targeting and reduced off-target effects, which are essential for advancing treatment methodologies that focus on precision medicine [54].

One promising biogenic approach involves utilizing exosomes as nanocarriers. Exosomes are naturally occurring vesicles secreted by cells that can mediate intercellular communication and transport biomolecules. Their ability to escape the immune response and their natural compatibility with biological systems make them excellent candidates for drug delivery systems [55,56]. Recent studies have illustrated the potential of exosomes as carriers for immunotherapeutics targeting ovarian cancer, effectively boosting therapeutic responses while minimizing systemic toxicity [55]. The integration of exosome technology into biogenic nanocarrier development could revolutionize delivery methods, enhancing the therapeutic landscape surrounding ovarian cancer treatment.

However, challenges persist in the application of biogenic nanoparticles, particularly in understanding their in vivo behavior. Despite advances in synthesis and characterization, the biodistribution, clearance rates, and interactions of biogenic nanoparticles within the tumor microenvironment remain areas of active research. Developing standardized protocols for assessing these factors is crucial to ensure the safety and efficacy of biogenic nanocarriers prior to clinical implementation [57,58]. Furthermore, the scalability of producing biogenic nanoparticles poses another challenge; ensuring consistent quality during large-scale production must be addressed to transition from research to clinical application [57,58].

## 3. Applications of Nanoparticles in Ovarian Cancer Diagnostics

**Figure 3:**
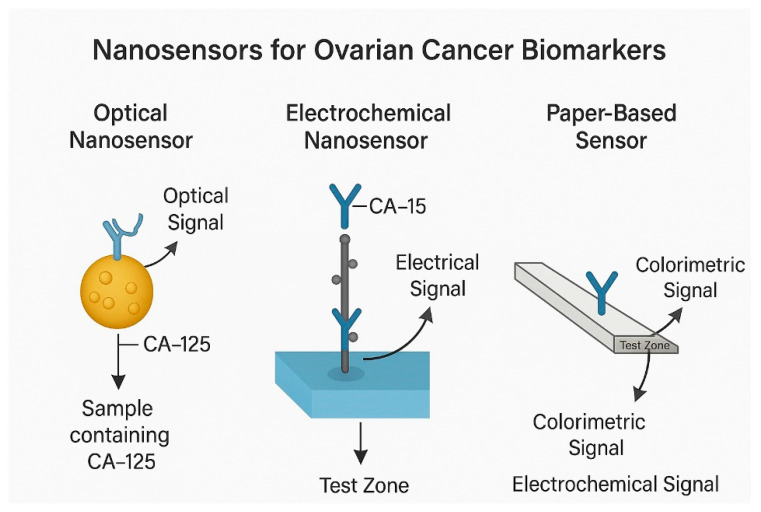
Nanosensor platforms for ultrasensitive detection of the ovarian cancer biomarker CA-125.

The identification of ovarian cancer poses challenges due to the vague symptoms that are prevalent in the initial phases of the illness, which can easily be confused with milder health issues [59]. Timely identification of ovarian cancer is vital as it significantly enhances the prognosis and boosts the likelihood of successful treatment. Symptoms such as bloating, abdominal discomfort, a sensation of fullness after meals, frequent urination, or changes in the menstrual cycle are often downplayed [60]. Furthermore, existing diagnostic techniques, such as the assessment of tumor markers (for instance, CA-125), ultrasound, computed tomography, and magnetic resonance imaging, are not consistently sensitive or specific enough to detect ovarian cancer in its early stages [7,61]. Early-stage cancer (stage I or II) indicates that the malignancy is limited to the ovaries or adjacent tissues. This stage enables effective surgical procedures and the initiation of chemotherapy. In such instances, survival rates are significantly higher in comparison to late-stage cancer (stage III or IV), when the malignancy has disseminated to distant organs [62]. Consequently, the advancement of more sensitive and specific diagnostic techniques using nanotechnology is crucial for enhancing outcomes in ovarian cancer [63]. Nanodiagnostics in ovarian cancer is likely to be pivotal in early detection, more accurate monitoring, and tailored therapies by utilizing cutting-edge nanotechnology to pinpoint biomarkers and molecular changes unique to ovarian cancer (Figure 3).

## 4. Types of Nanoparticles Used in the Study on Ovarian Cancer Treatment

### 4.1. Optical Nanosensors

**Figure 4:**
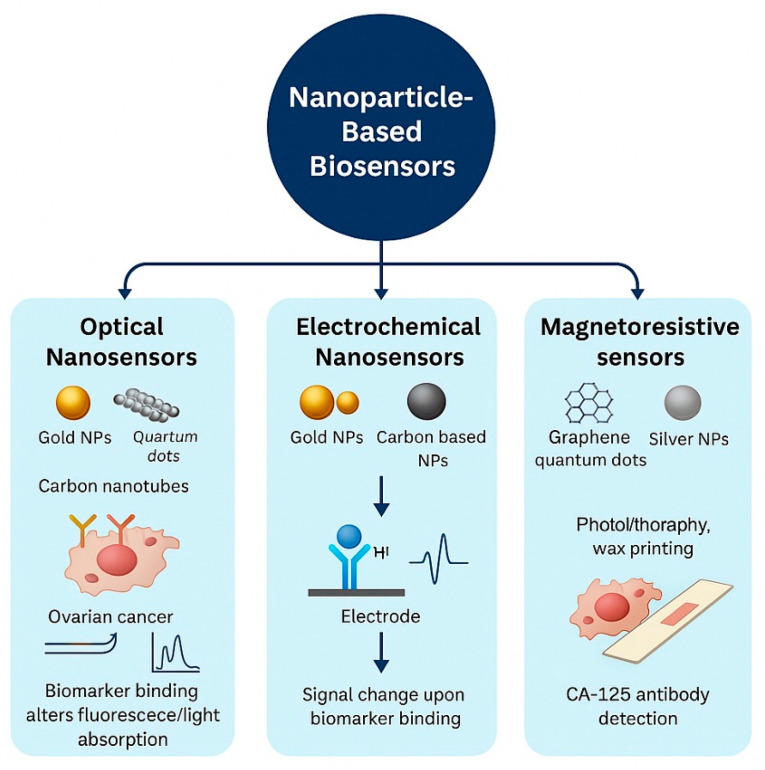
Nanoparticle imaging approaches for ovarian cancer detection.

Optical nanosensors are thought to possess transformative capabilities in the early diagnosis of ovarian cancer owing to their exceptional sensitivity and specificity [64,65,66,67]. These sophisticated nanosensors are capable of detecting subtle molecular alterations indicative of ovarian cancer in its initial stages [68]. Gold nanoparticles, along with quantum or carbon nanotubes, are utilized in the fabrication of these nanosensors. They are engineered to selectively attach to biomarkers found in the tumor microenvironment [53,69]. Consequently, due to their remarkable sensitivity and specificity, optical nanosensors can identify even minimal quantities of cancer biomarkers, rendering them rapid and precise tools for cancer diagnosis. Furthermore, their compatibility with high-resolution imaging techniques may facilitate the detection of small cancerous lesions that conventional methods might overlook. Additionally, the capacity of optical nanosensors to bind to cancer biomarkers results in alterations to their optical characteristics, such as fluorescence or light absorption [62], enabling the identification of cancerous lesions through spectroscopic techniques at the cellular level (Figure 4).

### 4.2. Electrochemical Nanosensors

Electrochemical nanosensors offer a viable method for accurately identifying biomarkers associated with ovarian cancer [70]. They are capable of detecting trace levels of biomarkers and various analytes [71]. Their functionality relies on electrochemical reactions that produce electrical signals corresponding to the concentration of a specific biomarker [72,73]. This process involves immobilizing a bioreceptor on the electrode’s surface. Within the biosensor framework, the bioreceptor identifies explicitly and binds to a designated cancer biomarker. After the biomarker attaches to the bioreceptor, a variation in the electrical signal strength that correlates to the quantity of bound biomarkers is recorded. These variations are quantified and analyzed, enabling precise identification of both the presence and amount of a biomarker within a biological sample. In the context of ovarian cancer, electrochemical biosensors can identify biomarkers such as CA-125, HE4, and other cancer-specific molecules [74]. Their exceptional sensitivity facilitates the diagnosis of the disease at early stages, thereby enhancing the likelihood of successful treatment and improving the prognosis for patients who have ovarian cancer [75,76].

### 4.3. Magnetoresistive and Paper-Based Biosensors

A distinctive benefit of giant magnetoresistive biosensors is their ability to detect several biomarkers at once [77], their exceptional precision, and their straightforward biomolecular identification [78]. Manufacturing processes such as photolithography and wax printing are employed in their creation [79,80,81]. The matrix construction method incorporates graphene-based quantum dots that are stabilized with silver nanoparticles. Consequently, to identify the CA-125 biomarker associated with ovarian cancer, research is currently focused on paper nanosensors that function by capturing the anti-CA-125 antibody on the surface of the nano matrix [82,83,84]. Thus, biosensors, whether they are optical, electrochemical, magnetoresistive, or paper-based, present novel strategies for diagnosing and tracking ovarian cancer. Nonetheless, none of the existing nanotechnologies achieves complete efficacy, and their practical use necessitates additional investigation. It is anticipated that with advancements in research and technological innovation, their significance in ovarian cancer diagnosis will expand, providing enhanced clinical alternatives.

## 5. Nanotherapy-Drug Carriers Based on Nanoparticles

Among the various nanoparticle drug carriers are liposomes, dendrimers, polymers, exosomes, and magnetic particles. Liposomes provide an effective means to deliver chemotherapeutic agents directly to ovarian cancer tumor cells. A notable example of such a carrier is liposomal doxorubicin (Doxil), which is employed in the treatment of recurrent ovarian cancer [85,86]. Liposomes protect the drug from degradation, allowing it to remain in circulation for a more extended period and enhancing its concentration at the tumor site [87,88]. Dendrimers consist of branched structures capable of carrying multiple drug molecules [89]. In ovarian cancer therapy, dendrimers are utilized as carriers for paclitaxel, a widely used drug in this context [90].

Additionally, dendrimers can be modified to target specific receptors located on the surface of cancer cells [91]. Polymers like polylactide-coglycolide (PLGA) are also applicable for the controlled release of anticancer medications in ovarian cancer cases [92,93]. Polymeric nanoparticles can release drugs in response to particular conditions in the tumor microenvironment, such as changes in pH or the presence of specific enzymes. An example of a nanoparticle demonstrating improved efficacy and fewer side effects compared to other polymers is paclitaxel carried by PLGA [93,94]. Gold nanoparticles can be employed in the photothermal therapy of ovarian cancer, which utilizes infrared light [95].

**Figure 5:**
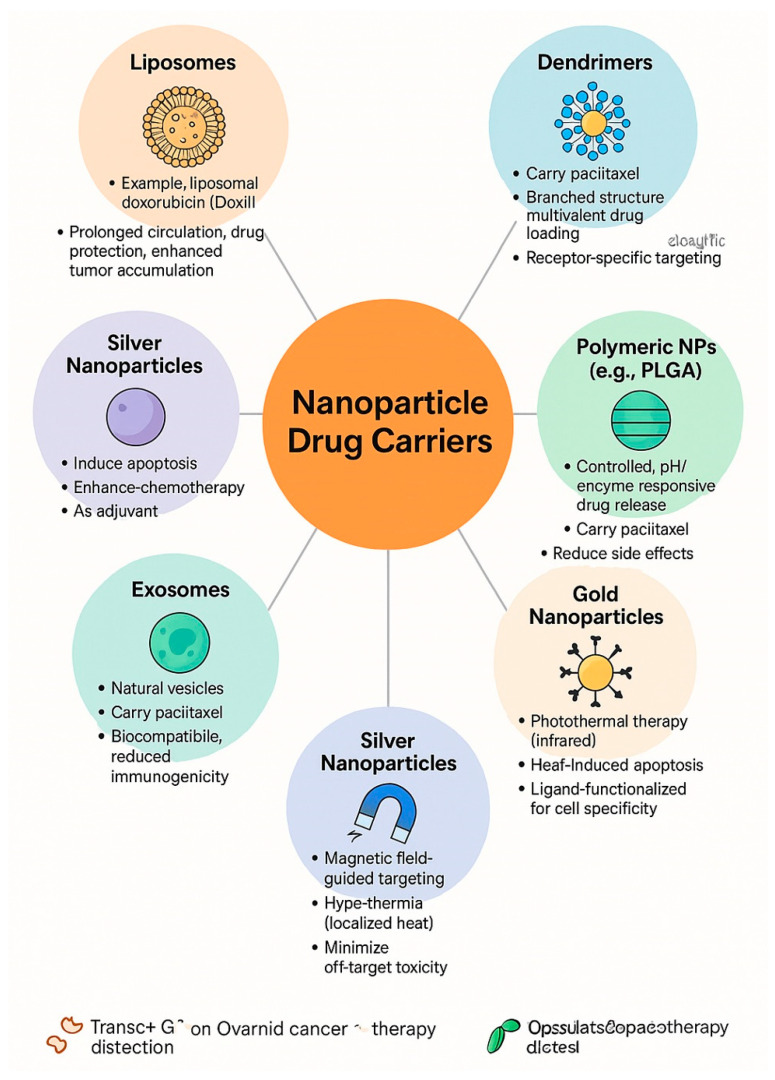
Nanoparticle drug carriers used in ovarian cancer therapy.

When subjected to infrared radiation, gold nanoparticles elevate their temperature, producing heat that inflicts damage and leads to the death of cancer cells. This method facilitates the targeting of tumors while minimizing harm to surrounding healthy tissue. Moreover, gold nanoparticles can be tailored by coating them with specific ligands, allowing them to target cancer cells with greater accuracy. Such modifications enhance the selectivity of the treatment, allowing it to more effectively eliminate only cancerous cells without impacting healthy ones [96]. Silver nanoparticles, recognized for their antibacterial properties, also exhibit anticancer effects by inducing apoptosis in ovarian cancer cells [97]. They can serve as adjuvants in chemotherapy, boosting the efficacy of drugs and enhancing their overall effects [98]. Magnetic nanoparticles can be utilized for targeted drug delivery in ovarian cancer cases [99,100]. An external magnetic field enables precise targeting of nanoparticles to the tumor, reducing damage to healthy tissues. Magnetic nanoparticles can also be involved in hyperthermia, a technique that heats nanoparticles to achieve localized destruction of cancer cells [101,102,103]. In summary, nanoparticle-based drug carriers hold great promise for the treatment of ovarian cancer (Figure 5). They facilitate targeted drug delivery directly to cancer cells, a factor that significantly enhances the effectiveness of the therapy [104,105].

The use of exosomes as drug delivery vehicles in ovarian cancer treatment shows considerable promise due to their unique biogenic characteristics, which confer distinct advantages over traditional drug delivery systems. Exosomes are lipid bilayer-enclosed vesicles that facilitate intercellular communication by carrying proteins, lipids, and RNA species, thereby influencing various biological processes. This capability has positioned them as potential carriers for anticancer drugs, particularly those targeting ovarian cancer.

The research has shown that exosome-loaded paclitaxel can significantly enhance drug accumulation within tumor cells, suggesting improved therapeutic efficacy against ovarian cancer compared to traditional synthetic nanoparticle delivery systems [106,107]. Models of ovarian cancer with exosomes encapsulating paclitaxel have demonstrated substantial induction of apoptosis, indicating their capability to deliver drugs effectively [107].

## 6. Benefits and Challenges of Nanoparticle Application (Figure 6)

Nanoparticles show great potential as alternatives in ovarian cancer treatment, yet they encounter various obstacles. A primary issue is their toxicity. When nanosystems deposit in the lungs, they can cause inflammation, oxidative stress, and cytotoxicity [108,109,110,111,112]. Moreover, healthy cells can be harmed by free radicals produced by nanoparticles [113]. Creating safe nanoparticles for human use is a complicated endeavor that requires several critical steps and methods [114,115].

**Figure 6:**
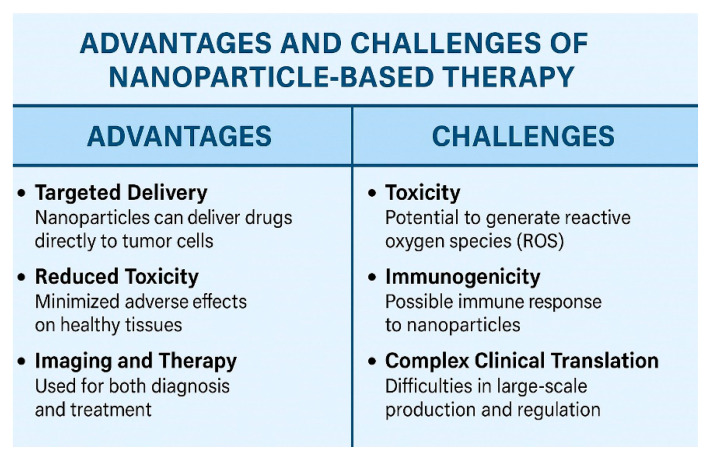
Advantages and Challenges of Nanoparticle-Based Therapy in Ovarian Cancer.

The initial step involves choosing biocompatible materials, which can include natural polymers, synthetic polymers, and metallic nanoparticles. Biodegradable and biocompatible natural polymers, such as chitosan, alginate, or hyaluronic acid, are ideal for medical applications [116,117]. Commonly, synthetic polymers like polyethylene glycol (PEG) are employed to enhance the surface of nanoparticles, improving their biocompatibility [118]. Additionally, metallic nanoparticles, such as gold and silver, are appreciated for their stability and biocompatibility [119]. The subsequent step is to coat the nanoparticles [120]. PEGylation, which involves covering nanoparticles with PEG, reduces their immunogenicity and extends their circulation time in the bloodstream, facilitating more efficient drug delivery to cancer cells [121]. Coating nanoparticles with other biocompatible substances, such as proteins or peptides, can also enhance their specificity towards cancer cells and minimize the likelihood of immune responses [122].

Furthermore, nanoparticle surface engineering can manipulate their interactions with cells and tissues. By functionalizing nanoparticle surfaces with ligands like antibodies or peptides, it becomes possible to bind to receptors on cancer cells selectively, thereby enhancing drug delivery accuracy [123]. It is crucial to conduct preclinical and clinical trials to assess the safety and effectiveness of the nanoparticles employed. In these studies, the biodistribution, toxicity, metabolism, and excretion of nanoparticles are thoroughly examined. The findings from these investigations will inform the design of nanoparticles optimized to minimize side effect risks and ensure safety in clinical contexts. For drugs to be accurately delivered to cancer cells, nanoparticles must successfully reach the intended site and release their therapeutic content in a controlled fashion. However, this process is hindered by the solubility of drugs, particularly hydrophobic ones, which can complicate their delivery [124]. This challenge can be addressed through lipid carriers, such as liposomes and lipid nanoparticles, which can encapsulate hydrophobic drugs, thereby enhancing their solubility and bioavailability [125]. Polymeric carriers, such as polymeric nanoparticles, microspheres, and dendrimers, facilitate controlled drug release and improve drug solubility. The strategies mentioned earlier enable precise drug delivery to targeted areas, enhancing therapeutic effectiveness while minimizing adverse effects.

A critical aspect of utilizing nanoparticles in ovarian cancer is the emphasis on targeted delivery systems, particularly those that exploit specific biomarkers prominently expressed in ovarian cancer cells. For instance, folate receptors (FRs) are often overexpressed in ovarian cancer, providing a strategic target for folate-conjugated nanoparticles [126,127,128]. These studies indicate that nanoparticles functionalized with folic acid achieve better targeting capabilities and enhanced delivery efficiency than non-targeted counterparts, thereby improving therapeutic efficacy against cancer cells such as SKOV3 [128,129]. Enhanced cytotoxicity has been observed with these targeted nanoparticles, representing an effective strategy to reduce systemic toxicity and improve clinical outcomes [126,130].

In various studies, researchers have reported successful in vivo applications of nanoparticle systems, emphasizing their role in chemotherapeutic drug delivery and synergistic effects with established agents such as paclitaxel and cisplatin. The effectiveness of utilizing polymeric nanoparticles co-delivering small interfering RNA (siRNA) has gained attention for overcoming multidrug resistance (MDR), a significant barrier in ovarian cancer therapy [131,132,133]. Agents like paclitaxel, when encapsulated within nanoparticles and accompanied by MDR-targeting siRNA, have demonstrated a marked increase in cytotoxicity towards resistant ovarian cancer cell lines, thereby addressing one of the most pressing challenges in treatment [134]. Nonetheless, despite promising preclinical results, the translation of these findings into clinical success remains a complex undertaking, often impeded by issues of bioavailability and systemic toxicity [135,136].

In terms of treatment outcomes, studies have reported varying success rates with nanoparticle-mediated therapies. While some reports showcase improved survival rates and tumor shrinkage in preclinical models [126,137,138], the results from clinical trials have not universally mirrored these findings, often due to the difficulty in achieving effective drug concentrations at target sites and responses to treatment varying significantly among patients [139,140]. A recent comprehensive review highlighted that, despite the introduction of promising nanoparticle technologies, only a few had received regulatory approval for clinical use, underscoring the need for ongoing research and validation [16].

The limitations of nanoparticle therapies extend to the complexity of biological systems, wherein factors such as tumor heterogeneity and the tumor microenvironment play critical roles in treatment responses. Resistance mechanisms among cancer cells, particularly regarding drug delivery modalities, present significant complications, as tumor cells often alter their metabolic pathways in response to chemotherapeutics [52,141]. Coupled with this are complications resulting from systemic circulation interference, as recent studies indicate that nanoparticles can accumulate in non-target tissues, potentially leading to adverse effects such as toxicity or compromised fertility, particularly in female patients undergoing treatment [142,143].

Despite facing various hurdles, the clinical application of nanoparticles presents several challenges. An increasing number of clinical trials contributes to the development of effective and safe nanosystems. Nonetheless, our current understanding of the safety of nanocarriers remains inadequate. To address this research gap, preclinical animal studies are essential for uncovering the risks associated with nanoparticle use, particularly concerning the body’s elimination processes [144].

One major regulatory hurdle lies in the characterization of nanomaterials. Unlike conventional drugs, nanoparticles exhibit unique biological behaviors that necessitate novel characterization techniques tailored to their nanoscale dimensions [145,146]. The National Cancer Institute (NCI) has undertaken initiatives to develop standardized protocols for the characterization of nanomaterials, emphasizing the need for comprehensive evaluation concerning their pharmacokinetics, biodistribution, and potential toxicity [146,147]. The NCI’s Nanotechnology Characterization Laboratory plays a pivotal role in collaborative efforts with regulatory agencies, striving to build a cohesive framework that guides the development process from bench to bedside [146].

However, traditional regulatory frameworks may not adequately capture the nuances of nanoparticle-based therapies, necessitating the development of new guidelines or amendments to existing regulations to ensure that the evaluation process is reflective of the unique characteristics of nanotechnology [148]. The variability in international regulatory standards further emphasizes the need for harmonization to streamline the approval process across jurisdictions and facilitate the global implementation of nanotechnologies in ovarian cancer treatment [149].

Additionally, increasing collaboration between regulatory agencies and academic institutions is essential to foster innovation while ensuring patient safety. By staying engaged with researchers and developers, regulatory bodies can gain insights into emerging technologies and anticipate potential challenges within the clinical translation pipeline [31,150]. This synergistic approach to innovation can ultimately facilitate regulatory approvals while ensuring robustness in patient safety and therapeutic efficacy.

However, despite these challenges, nanoparticles present considerable opportunities. They have the potential to significantly diminish the side effects of chemotherapeutic agents, increase drug delivery specificity, and enhance drug solubility. In the realm of diagnostics, nanotechnology could lead to the creation of portable nanosensors suitable for use outside clinical environments, which is vital for the early detection of ovarian cancer. Additionally, the development of nanosensors paves the way for new commercialization opportunities for biosensors, which could foster broader adoption of these technologies in clinical practice.

For example, a significant success story is the development of Doxil (liposomal doxorubicin), which illustrates the practical application of nanotechnology in cancer therapy. Approved for treating recurrent ovarian cancer, Doxil exemplifies how nanoparticles can enhance drug solubility, reduce toxicity, and improve therapeutic efficacy. Clinical trials have shown that Doxil increases progression-free survival in women with platinum-sensitive ovarian cancer, thus demonstrating the tangible benefits of nanoparticle formulations in clinical settings [150,151]. This success has paved the way for further innovations in nanomedicine, setting a precedent for future therapies that utilize similar nanoparticle delivery systems.

Despite this success, various challenges impede the clinical application of nanoparticle technologies. A primary issue is the complexity and heterogeneity of ovarian cancer tumors. The tumor microenvironment often differs significantly from preclinical models used during initial research, creating discrepancies between anticipated outcomes and actual therapeutic efficacy in patients [152,153]. The diversity among ovarian cancer subtypes means that while nanoparticles may be effective for one type, they could be ineffective for another, necessitating a more personalized approach that includes comprehensive biomarker analysis to ensure proper patient stratification for nanoparticle therapies [154].

Therefore, advancing and refining nanoparticles is essential to unlock their full potential in both diagnosis and treatment.

## 7. Summary

The integration of nanoparticles in ovarian cancer management represents a significant advancement, potentially leading to breakthroughs in early detection and the development of personalized therapeutic strategies.

Various types of nanoparticles, including iron oxide, gold, silver, semiconductor, organic, carbon, silica, and fullerenes, possess unique properties that are advantageous for cancer diagnosis and therapy and are currently under investigation. Advanced nanosensors, including optical, electrochemical, and paper-based types, are being developed to detect biomarkers of ovarian cancer. These sensors offer high sensitivity and specificity, intending to improve early diagnosis and personalized treatment approaches (Table 1). Nanoparticle drug carriers such as liposomes, dendrimers, and gold nanoparticles provide effective drug delivery mechanisms that enhance the bioavailability of therapies while reducing side effects.

However, challenges remain, including potential toxicity, biocompatibility issues, and the necessity for extensive clinical testing. Ongoing research is crucial for addressing these challenges and fully harnessing the potential of nanoparticles in the treatment of ovarian cancer.

One of the most significant trends in developing nanoparticles for ovarian cancer treatment is the emphasis on targeted drug delivery systems. Folate-conjugated nanoparticles, for example, have been researched extensively due to their ability to exploit the overexpression of folate receptors present in many ovarian cancer cells. Studies show that these nanoparticles can improve drug uptake and enhance therapeutic outcomes compared to conventional drugs [90,126]. As research progresses, there will be a greater push for multifunctional nanoparticles that can deliver not just chemotherapeutics but also molecularly targeted agents, such as siRNA or nucleic acids, to silence specific oncogenes and reverse drug resistance mechanisms often seen in ovarian cancer [134,155].

Additionally, theranostic nanoparticles that combine therapeutic and diagnostic capabilities are making headway. This dual functionality allows for real-time monitoring of treatment responses and tumor dynamics, potentially leading to more personalized cancer treatment strategies. For instance, HER2-targeted theranostic nanoparticles have shown promise in treating heterogeneous ovarian cancers while providing imaging capabilities to track treatment efficacy [137]. The integration of imaging agents with therapeutic nanoparticles will likely become a notable trend, enhancing the ability to tailor treatment regimens based on tumor responsiveness over time.

Moreover, the use of innovative nanoparticle materials—such as gold nanoparticles, silica nanocarriers, and dendrimers—is expanding. Gold nanoparticles, for instance, have been explored for their cytotoxic effects against ovarian cancer cells and their potential to serve as carriers for chemotherapeutics [156,157]. The tunable properties of these materials enable scientists to modify their size, shape, and surface chemistry to achieve optimal interaction with cancer cells rather than healthy cells. This adaptability is crucial for minimizing side effects and enhancing patient outcomes.

Emerging technologies, such as RNA-based nanoparticles, represent another front in the fight against ovarian cancer, particularly in addressing issues of multidrug resistance (MDR). Nanoparticles encapsulating siRNA to target genes responsible for MDR have shown significant promise in both preclinical and clinical settings, illustrating that targeted gene knockdown can effectively improve the sensitivity of cancer cells to chemotherapy [131,158]. Future research will likely continue to explore innovative ways to enhance the efficacy of RNA therapeutics through improved delivery mechanisms, thereby transforming the landscape of ovarian cancer treatment.

## Figures and Tables

**Table 1:** Different platforms of nanoparticles used in a study on the treatment of ovarian cancer.

Feature	Liposomes	Polymeric NPs	Dendrimers	Gold NPs	Iron Oxide NPs
Structure	Phospholipid bilayer vesicles	Biodegradable polymer matrix	Highly branched synthetic polymers	Inorganic metallic core	Superparamagnetic iron oxide core
Drug Loading	Hydrophilic (core) + lipophilic (bilayer)	Hydrophobic/hydrophilic (tunable)	Surface or internal cavity loading	Surface adsorption/conjugation	Surface or encapsulated drugs
Targeting Potential	High (PEGylation, ligand-conjugation)	High (modifiable surface)	Very high (multiple functional end-groups)	High (easy to conjugate targeting ligands)	High (can be guided magnetically or with ligands)
Biocompatibility	Excellent	Good to excellent	Moderate to good	Variable (depends on size/surface modification)	Good (FDA-approved for imaging)
Imaging Capability	Limited	Limited	Limited	Excellent (CT, photoacoustic)	Excellent (MRI contrast agent)
Theranostic Use	Moderate (drug + limited imaging)	Moderate	Moderate	High (therapy + imaging)	High (hyperthermia + imaging + drug delivery)
Clinical Trials	Several in advanced stages	Some ongoing trials	Preclinical/early stage	Mostly preclinical	Some in imaging, early therapy trials
Challenges	Stability, RES uptake	Burst release, scaling production	Toxicity at higher generations, synthesis complexity	Long-term toxicity, accumulation in organs	Heating control, clearance from the body
